# Methicillin-Resistant *Staphylococcus aureus* Diabetic Foot Crossed Infection: A Case Report

**DOI:** 10.3390/pathogens9070549

**Published:** 2020-07-08

**Authors:** María Reina-Bueno, Inmaculada C. Palomo-Toucedo, Aurora Castro-Méndez, Gabriel Domínguez-Maldonado, María del Carmen Vázquez-Bautista

**Affiliations:** Department of Podiatry, University of Seville, 41009 Seville, Spain; ipalomo@us.es (I.C.P.-T.); auroracastro@us.es (A.C.-M.); gdominguez@us.es (G.D.-M.); carmenvaz@us.es (M.d.C.V.-B.)

**Keywords:** diabetic foot, diabetic foot infection, foot ulcer, methicillin-resistant *Staphylococcus aureus*, healthcare-associated infection, multidrug-resistant infections

## Abstract

This work presents a protocol to prevent the transmission of multidrug-resistant infections. We focus on the Diabetic Foot Unit Podiatry Clinic Area attached to the University of Seville in particular. The most common complication for patients with diabetes is leg ulcers. Together with neuropathy, vasculopathy, and immunological response disorder, these individuals have a high predisposition to developing infections. Staphylococcus aureus is a highly prevalent microorganism in humans which, at times, may act as a pathogen. Due mainly to indiscriminate abuse of antibiotics, the methicillin-resistant strain known by its initials as MRSA is the most extended nosocomial infection globally and is a severe community and hospital healthcare problem. This paper describes compliance with new general recommendations on cleaning, hygiene, and decontamination, in addition to implementation of this specific protocol, after detection of cross infection (healthcare-related infection) in the studied unit in two patients with MRSA-infected ulcers. After an in-depth bibliographical review, strict hand hygiene measures and use of non-sterile gloves were used when treating all patients with a diabetic foot. Finally, we reflect on the need to educate healthcare personnel to guarantee correct prescription of selected antibiotics. The role of the podiatrist in the multidisciplinary team is highlighted not only in terms of management and treatment of lesions in diabetic patients, but also as a healthcare agent for the detection and prevention of MRSA together with other multidrug-resistant infections.

## 1. Introduction

Diabetes mellitus (DM) is a combination of metabolic disorders characterized by hyperglycemia where insulin is incapable of functioning and/or the amount of segregated insulin is insufficient to obtain the necessary hypoglycemic effect. In 2019, 463 million people were diagnosed with DM globally. However, the prevalence of this disease is on the rise. It is estimated that in 2045 there will be approximately 700.2 million people with DM [1].

Diabetic foot syndrome is defined by the World Health Organization (WHO) as “ulceration, infection, and/or gangrene of the foot associated with peripheral neuropathy in different degrees of peripheral arterial disease and the result of the complex interaction of different factors” [2]. 

These ulcers are the consequence of a complex interaction of several pathophysiological factors, mainly neuropathic, vasculopathic, and immunopathic. Approximately 15% to 25% of patients with DM develop diabetic foot ulcers (DFUs) over the course of their lifetime [3]. This leads to a major socioeconomic impact and loss of the patient’s quality of life. This condition is more common in men than in women [4].

Over 50% of these ulcerations will become infected, resulting in high rates of hospitalization, increased morbidity, and potential lower limb amputation. Predisposing factors for the development of diabetic foot infection (DFI) in diabetic patients include neuropathy, vasculopathy, and immunopathy [5].

DFI treatment requires wound care, antimicrobial therapy, and often surgical procedures. As a result, DFIs are the most common diabetic complication requiring hospitalization and the world’s leading cause of nontraumatic lower limb amputation [3].

Several studies have revealed that DFIs are polymicrobial, including bacterial flora (*Staphylococcus aureus*, *Streptococcus epidermidis*, Peptococcus, and *Bacteroides fragilis*) [6,7]. *Staphylococcus aureus* is the most commonly isolated microorganism [6,7,8,9], but the extended spectrum beta-lactamases Klebsiella and *Pseudomonas aeruginosa* can also be detected in small concentrations, increasing the risk of limb amputation [6,7,8].

As with other transient flora bacteria, *Staphylococcus aureus* is both a commensal bacterium and a human pathogen [3,9]. Approximately 15–30% of the human population is colonized with *Staphylococcus aureus* [9]. Nevertheless, according to the meta-analysis by Stacey et al. patients with diabetes have a 4.75% higher colonization rate than non-diabetic people [10]. Importantly, this bacterium causes a wide range of clinical infections (e.g., bacteremia, endocarditis, skin and soft tissue, osteoarticular, pulmonary, and device-related infections). The prevalence of methicillin-resistant *Staphylococcus aureus* (MRSA) in DFI is 16.78% [10]. However, subsequent studies have reported higher rates in accordance with geographic variables [6,7,9].

The most important mechanism of the propagation of MRSA and the other microorganisms cited below is contact between people. The most common transfer is from one person to another by means of contaminated hands of healthcare/auxiliary personnel who do not wash them correctly between one patient and the next [8]. Transmission because of contamination of healthcare material and/or surfaces has also been reported and is known as healthcare-associated infection (HAI). Hygiene and disinfection guidelines are essential for prevention in the hospital and healthcare center settings. To avoid these infections, a series of standard precautions is necessary for all patients, and some specific regulated procedures must be added in those patients diagnosed with MRSA in general as well as those with DFI [11,12,13].

The Diabetic Foot Unit of the Podiatry Clinic Area attached to the University of Seville has a care, research, and teaching purpose, because it sees patients, performs various research lines, and is a location for work experience for students completing a graduate degree in podiatry. This unit provides diabetes mellitus patients with full preventive care and treatment. Seventy percent of consultations are for ulcer management. In June 2018, under clinical suspicion of wound infection, ulcer sampling was conducted in several cases. Microbiological cultures confirmed for two patients presented MRSA in DFI. Cross infection had occurred, so HRI was diagnosed. In these cases, in addition to the standard precautions carried out, a specific protocol is set out below with the purpose of stopping transmission as a part of a multimodal strategy. This study was authorized by the Research Ethical Committee of the University Hospitals Virgen Macarena and Virgen del Rocío. Written consent was obtained from the participants. 

## 2. Case Presentation

### 2.1. Case 1 (Possible Imported Hospital Case) 

A 68-year-old retired diabetic woman.

Family history: Unremarkable.

Personal history: No known allergies, insulin-dependent type 2 diabetes mellitus, aged 25 with well-controlled clinical symptoms. The patient was on metformin (500 mg) two daily before breakfast and after dinner. Pre-prandial glucose was 160 mg/dL and HbA1c was 8%. Well-controlled hypertension under treatment with atenolol; high cholesterol under treatment with fluvastatin; former smoker; being treated with platelet anti-aggregant. Amputation of proximal phalanx of the first left toe 2 years ago. Discharged from hospital 3 weeks ago after presenting with acute pyelonephritis and bacteremia. 

Presented a neuro-ischemic ulcer with a 2-week clinical course under the heads of the second and third metatarsals of the left leg with a foul smell and abundant exudate. This infection was classified as grade 2 [14]. Biofilm was not detected.

Examination: This revealed pale coloring of the skin with coldness. Neurological examination revealed no sensitivity with the Semmes–Weinstein monofilament and Rydel–Seiffer tuning fork. The ankle–brachial index revealed arterial calcification and rigidity in the joints. A probe to bone (-) was done.

Additional tests: Dorsoplantar X-ray did not detect any signs of osteoarthritis. A sample was taken by swab for microbiological culture and methicillin-resistant *Staphylococcus aureus* was isolated.

Treatment: Cefuroxime (500 mg/12 h) was prescribed according to an antibiogram, and the ulcer was treated twice per week according to the TIME algorithm: [15,16,17].

- The wound was cleaned and decontaminated with a solution that is also effective for MRSA (purified water, 0.1% undecylamidopropyl betaine, and 0.1% polyhexamide);
- Debridement of devitalized tissue and slough;
- Protection of peri-lesional tissue with a non-irritant skin protector;
- Carbon and silver dressing;
- Alginate dressing;
- Polyurethane foam dressing.

This treatment recommendation was maintained for 4 months and these dressings were subsequently replaced by metalloproteinase modulator matrix for 25 days until full healing. After full healing, pre-prandial glucose was 90 mg/dL and HbA1c was 6.8%.

During this time, due to having a notifiable disease, the patient continued to be reviewed in the Departments of Infection and Endocrinology in their reference hospital until infection was eradicated.

### 2.2. Case 2

A 48-year-old retired man with total permanent incapacity.

Family history: Father with type 2 diabetes mellitus, Alzheimer’s disease, and a mother with Alzheimer’s disease.

Personal history: No known allergies, poorly controlled type 1 diabetes mellitus with a clinical course of 20 years. The total daily insulin dose was 24 UI (breakfast 8 UI; lunch 6 UI; dinner 8 UI). Pre-prandial glucose was 180 mg/dL and HbA1c was 9%. Well-controlled hypertension under treatment with losartan; high cholesterol under treatment with simvastatin; smokes 15 cigarettes a day. Transmetatarsal amputation of the left foot 8 years ago. 

Presented with a neuroischemic ulcer of with a clinical course of 3 months that appeared after a blister-like lesion in the dorsal area of the fifth toe of the right foot. This was classified as a grade 3 infection [14] and treated with Betadine. Biofilm was not detected. Given that the clinical course of the patient was incomplete, their podiatrist referred him to our center for follow-up.

Examination: Inspection revealed good coloring on both sides of the leg and no hair. Neurological examination did not reveal any sensitivity to the Semmes–Weinstein monofilament and Rydel–Seiffer tuning fork. The ankle–brachial index was 0.9, and joint rigidity was shown. A positive probe to bone in the head of the middle phalanx of the 5th toe of the right foot was found. 

Additional tests: Dorsoplantar X-ray revealed signs of osteomyelitis. A swab sample for microbiological culture gave a positive result for methicillin-resistant *Staphylococcus aureus*.

Treatment: Oral vancomycin (1000 mg/12 h) was prescribed according to an antibiogram and the ulcer was treated twice a week according to the TIME algorithm: [15,16,17].

- The wound was cleaned and decontaminated with a solution that is effective for MRSA (purified water, 0.1% undecylamidopropyl betaine, and 0.1% polyhexamide);
- Debridement of devitalized tissue and slough;
- Protection of peri-lesional tissue with non-irritant skin protector;
- Application of hydrogel to the wound bed;
- Nanocrystalline silver dressing;
- Polyurethane foam dressing.

This treatment was maintained for 1.5 months and these dressings were subsequently replaced with a metalloproteinase modulator matrix for 2 weeks up until fully healed. After full healing, pre-prandial glucose was 110 mg/dL and HbA1c was 7.5%.

During this time, due to having a notifiable disease, the patient continued to be reviewed in the Departments of Infection and Endocrinology in their reference hospital until infection was eradicated.

## 3. Proposed Protocol

According to the recommendations of the Spanish Network for Epidemiological Monitoring [13], all patients seen in the same consultation room or who shared the same therapeutic intervention were located. They were investigated, and no other patient was found to be infected by MRSA because of non-compliance with the two necessary requirements, which are a positive microbiological culture for this pathogen and signs of infection [11,12,13].

Standard precautions were followed with the aim to stop transmission between patients and to prevent cross-infections, even by means of fomites during the provision of podiatry care.

- The patient must be scheduled in the last timeslot for the day, wherever possible. It should be recorded that this is a patient in contact isolation.
- Treatment room carts should remain empty.
- Before the patient enters the room, all the furniture in the room should be covered with disposable cloths, which are exclusively used for patients in contact isolation.
- It is essential to wash hands with antiseptic soap and running water before and after seeing the patient. Before intervention, alcohol-based handrub (ABHR) may be used to prevent skin damage.
- Dry hands using disposable towels.
- The podiatrist must prepare the material necessary for treatment in advance.
- The patient must enter unaccompanied and leave their personal effects behind (jewelry, coats, jackets, crutches, pushchairs, etc.).
- Put the disposable material generated during the treatment into a container with a bag so that it does not remain on the floor and has no contact with the environment. Gauzes and remaining disposable material used should go directly into the usual containers which are processed normally. Surgical material that requires sterilization is processed normally.
- All personnel who take part in the process in some way must wear a disposable gown, overshoe covers, a cap, gloves, and a mask. Once the treatment is finished, all of this material should be removed and placed in the aforementioned container.
- Disposable material that covers the chair and remaining furniture should go in the container with the remaining contaminated tissues.
- The container’s bag must be sealed at the end.

Once the intervention is over, the room, including mobile objects, side tables, and clinical furniture (chairs, lamps, and stools), should be cleaned exhaustively with 1% sodium hypochlorite or concentrated disinfectant detergent (TP18). The materials necessary to perform this cleaning should be exclusively used for this kind of treatment and should undergo contact isolation so that they are only used in the treatment room. This must be performed from the cleanest areas to the areas with the most waste.

The floor should be cleaned by means of mopping with a damp mop in a zigzag direction. It should subsequently be cleaned with a mop and bleach in a 1:10 proportion (30 mL of common bleach per liter of water).

In parallel, some rules need to be implemented in the diabetic foot unit to manage all patients.

- The podiatrist must wash his hands at the start and end of his working day as well as before and after seeing each patient.
- Rings and bracelets must be removed to avoid the adherence of any substance or microorganism.
- The podiatrist must wear gloves when treating patients. These must be changed for each patient or if they break.
- The patient must enter unaccompanied.
- The podiatrist and students must use greeting words and refrain from shaking hands. After finishing the intervention, a farewell without direct contact is necessary.
- Complete disinfection of foot and ankle with antiseptic should be done, reaching the distal third of the leg to disinfect the skin close to the ulcer.
- Patients must receive hygiene education and promotion.
- Treatment carts must only have material that is essential for performing the treatments.
- At the end of each shift, the floor and surfaces should be cleaned and disinfected by the same procedure as outlined above.

In addition, The Podiatry Clinic Area implemented a multimodal strategy to improve the hand hygiene compliance in parallel. This intervention included four components recommended by the WHO with adaptations for this context: system change (number of sinks near of the point of care, access to ABHR dispensers), education (specific training sessions for all the staff, including cleaning personnel and students), reminders in the workplace (“how to handwash” and “my five moments for hand hygiene” posters), and safety climate (patient participation, DFI antibiotic treatment manual) [18].

## 4. Discussion

We have reported two MRSA case studies in patients from the Diabetic Foot Unit in the University of Seville. As reflected in the literature, diabetic foot infection is a complication that can affect 50% of diabetic patients and is related to high rates of morbidity and mortality [3].

According to the European Commission, HRI are infections that a person can get when receiving healthcare or during their stay in a treatment center (health center, hospital, day care center, geriatric center, etc.) and are not present in the incubation period at the time of receiving this treatment. These infections are favored when there are abnormalities in defense mechanisms, when the patients have a chronic disease such as DM, and also when there are interruptions in the continuity of skin, such as ulcers [11,19,20]. Diabetic patients have multiple risk factors for colonization with methicillin-resistant *Staphylococcus aureus* (MRSA), a nosocomial pathogen associated with significant morbidity and mortality rates [7,10].

Infections caused by multidrug-resistant microorganisms are a major challenge for treatment by the usual methods, mainly due to the biofilms formed by bacteria and the difficult management with antibiotherapy. It is for this reason that prevention is a fundamental pillar [3].

A large number of these infections occur in healthcare centers, so it is necessary to increase prevention and hygiene measures to avoid them [21,22,23,24,25,26]. Patients with this type of infection require two types of action, general and specific [13,19].

According to microbiological sampling, both infections described in this study were related, and we can state that cross infection occurred in our center as it was detected within a period of less than one month [3,8]. One of the most important procedures implemented in our unit is hand hygiene at the start and end of the working day and before and after seeing every patient. 

Hand washing is replaced by hand hygiene, which involves detailed and systematic washing to achieve effective decontamination of the skin [11,18,20,21,24,27]. We need to clarify the general measures set out by the WHO and collated in various guidelines which recommend the use of hydroalcohols [11,18,27] or chlorhexidine after managing MRSA patients when dirtiness is not observed [11,18,24]. This is due to three reasons. First, regarding the pathogen, *Staphylococcus aureus* is a bacterium that does not produce spores. Second, such compounds have been proven to be more effective as antiseptics [20,21,24], and third, as a work-related preventive measure because soaps are more likely to lead to skin abnormalities such as xerosis or dermatitis in healthcare workers and carers than ABHR [11,18,20,24,27,28].

ABHR should be used with a correct technique following WHO recommendations for optimal results [18]. The volume, extension, and time required for application are being studied. As a general rule, a sufficient amount of solution depending of the size of the hand, about 2–3 mL, is enough. Rubbing for 30 s to cover all surfaces on the palm, dorsum, all fingers, thumbs, and between them is required. 

Given that exposure to exudates, blood, or secretions often occurs in our diabetic foot department, it is preferable to perform this hygiene method with soap and water; although if there are no remnants of dirt, this could be performed with ABHR [27,29,30]. Solutions that contain 60% to 95% alcohol are the most effective [3]. This is the simplest, most effective, and most efficient method to prevent transmission during healthcare [11,31].

Because of this same circumstance, another rule implemented in our department is the obligation to treat all patients with non-sterile gloves. This rule is in response to the fact that in our unit there is direct contact with non-intact skin [29,30]. Wearing gloves must be a general requirement when treating all patients, because due to sensitivity problems, DM patients may present foot wounds that go unnoticed [2].

The use of gloves must not ever replace correct hand washing [24,31]. Wearing the same pair of gloves for more than one patient is an unacceptable technique, as washing gloves is not recommended. [20,27] They must be correctly disposed of after contact with any contaminated patient or surface [29,30]. On the contrary, incorrect use of gloves may potentially be responsible for infection [31], as reported by McBride et al. who recorded 17% transmission from patients colonized with MRSA from health workers’ gloves [2,32]. 

In the case of patients colonized or infected with MRSA, strict prevention measures are needed, so a protocol to treat these patients was established following recommendations by different guidelines [12,19,29] and papers [22,23,26,27].

Considering the high rate of general population MRSA colonization at the Diabetic Foot Unit Podiatry Clinic Area of the University of Seville, which is even higher in patients with diabetes mellitus [10,21,27,30], patients are cared for unaccompanied, except those that are dependent or under 18 years of age. Even though we have not found any specific recommendations in the guidelines, it remains clear that colonized patients can be vectors of the infection to others or can self-contaminate their own wound. The patient may have an active role in the transmission of these diseases [23]. Therefore, as a preventive measure, we recommend the avoidance of shaking hands. Istenes et al. reported that 39% of patients studied showed contamination from at least one pathogen on their hands and specifically 14% presented a positive MRSA culture, 48 h after hospital admission [26]. For this reason, promoting hand hygiene programs and good practices among patients, in general, can contribute to reducing HAI.

Different training sessions on hand hygiene are also held to prevent the spread of multiresistant organisms, as recommended by the WHO [18] and the prevention guidelines. All of our site’s personnel attend these activities [12,13,23,25,27].

These infections must be declared, and the patients must be followed up and monitored jointly with the department of infectious diseases and microbiology of Virgen Macarena University Hospital [12,13].

The podiatrist’s function within this multidisciplinary team not only lies in prevention, follow-up, and treatment of the diabetic foot, but they are also essential for the early detection of infectious processes. According to the literature, the identification of clinical signs of ulcer infection is the first step for the culture of wounds. Subsequent microbiological confirmation is what enables verification of suspicions [6,12,13]. 

Despite the fact that MRSA is usually sensitive to topical antibiotics, such as mupirocin or fusidic acid, it is preferable not to use them in patients with chronic ulcers such as FPI, because the pathogen easily becomes resistant to them. [8]. Thus, in our guidelines for managing diabetic ulcers, local antibiotic therapy is not presented. 

Detailed inspection and evaluation of lesions enables a distinction to be made between a contaminated and an infected ulcer [12,13,33]. Diagnosis must set out the criteria for correct treatment and antibiotics should only be used in case of infection and not as generalized prophylaxis. Selective antibiotic treatment, in addition to the patient’s adherence to this, is fundamental to treat and prevent this process and to avoid propagation of MRSA infection because prolonged and indiscriminate use of antimicrobials is related to increased multiresistant infections [6,12,13,34], which entails a threat to the global health system. [12,13,33,35] 

Healthcare personnel must update their knowledge to identify resistance patterns in the diabetic foot and improve their training based on guidelines on the correct use of antibiotics [6,12,13,33,35]. 
